# Comparative efficacy of different traditional mind-body exercises in patients with stable chronic obstructive pulmonary disease: a systematic review and network meta-analysis

**DOI:** 10.3389/fmed.2025.1678352

**Published:** 2025-10-10

**Authors:** Xiang Lin, Qianqian Xu, Wenshan Xu, Wen Sun, Hui Sun, Shungui Xu

**Affiliations:** ^1^Department of Respiratory Medicine, Affiliated People’s Hospital of Fujian University of Traditional Chinese Medicine, Fuzhou, Fujian, China; ^2^Fujian University of Traditional Chinese Medicine, Fuzhou, Fujian, China

**Keywords:** chronic obstructive pulmonary disease, traditional mind–body exercises, pulmonary function, exercise capacity, quality of life

## Abstract

**Objective:**

This study aimed to systematically compare the differential therapeutic effects of various traditional mind–body exercises (TaiChi, Liuzijue, Wuqinxi, Baduanjin, Yijinjing, and Yoga) in patients with chronic obstructive pulmonary disease (COPD) through a network meta-analysis, providing evidence-based support and a scientific rationale for developing individualized rehabilitation strategies.

**Methods:**

A comprehensive search was performed to identify randomized controlled trials (RCTs) investigating traditional mind–body exercise interventions in COPD patients. Databases including PubMed, Embase, Web of Science, Cochrane Library, CNKI, WanFang, and the VIP Database were searched from their inception to June 2025. The methodological quality of included studies was assessed using Cochrane Risk of Bias tool, while the network meta-analysis was conducted in Stata 14.0 using the frequentist model. Publication bias was assessed using funnel plots.

**Results:**

A total of 83 RCTs comprising 6,707 COPD patients were included. For improving the percentage of predicted forced expiratory volume in 1 s (FEV1%), the interventions ranked as follows: Wuqinxi (SUCRA = 82.9%) > Yijinjing (SUCRA = 65.9%) > Liuzijue (SUCRA = 63.5%) > Baduanjin (SUCRA = 63.1%) > Yoga (SUCRA = 47.8%) > TaiChi (SUCRA = 25.3%) > Conventional treatment (SUCRA = 1.5%). For enhancing the 6-min walk test (6MWT), the ranking was: Wuqinxi (SUCRA = 96.7%) > Baduanjin (SUCRA = 80.6%) > TaiChi (SUCRA = 53.1%) > Liuzijue (SUCRA = 49.1%) > Yoga (SUCRA = 42.9%) > Yijinjing (SUCRA = 26.8%) > Conventional treatment (SUCRA = 0.9%). For reducing the COPD Assessment Test (CAT) score, the ranking was: Yijinjing (SUCRA = 96.7%) > TaiChi (SUCRA = 75.9%) > Wuqinxi (SUCRA = 63.2%) > Baduanjin (SUCRA = 42.4%) > Yoga (SUCRA = 37.7%) > Liuzijue (SUCRA = 33.0%) > Conventional treatment (SUCRA = 1.2%).

**Conclusion:**

Wuqinxi demonstrated the greatest benefits in improving pulmonary function and exercise capacity, whereas Yijinjing showed the most significant effects in enhancing quality of life. Different mind–body exercise interventions may promote rehabilitation through distinct physiological and psychological mechanisms. Individualized selection of interventions can be tailored based on patients’ functional status and rehabilitation needs.

**Systematic review registration:**

https://www.crd.york.ac.uk/PROSPERO/view/CRD420251051806, identifier CRD420251051806.

## 1 Introduction

Chronic obstructive pulmonary disease (COPD) is a preventable and treatable chronic respiratory disorder characterized by persistent, partially irreversible airflow limitation and impaired lung function (1, 2). COPD can severely affect multiple systems, including the respiratory, muscular, psychological, and digestive systems, leading to reduced exercise capacity, diminished quality of life, and even mortality (3, 4). Epidemiological studies indicate that by 2017, the number of patients with chronic respiratory diseases was estimated at 544.9 million, with approximately 55% of cases attributable to COPD. Furthermore, by 2019, COPD had become the third leading cause of death worldwide (5, 6). Consequently, effective prevention and treatment of COPD remain pressing challenges for global public health.

Pulmonary rehabilitation is regarded as a cornerstone intervention for managing stable COPD, as it can effectively alleviate dyspnea, enhance exercise capacity, and improve quality of life. It is widely recommended by multiple international clinical guidelines. The joint clinical practice guidelines issued by the American College of Chest Physicians and the American Association of Cardiovascular and Pulmonary Rehabilitation emphasize that exercise training constitutes the core component of pulmonary rehabilitation, serving as a primary approach to reducing dyspnea, enhancing exercise tolerance, and improving health-related quality of life in COPD patients. It may also help slow the decline of lung function to some extent (7). However, conventional pulmonary rehabilitation modalities such as aerobic exercise and equipment-based training face challenges, including poor patient adherence and high resource demands (8).

In recent years, mind–body exercise interventions originating from traditional medicine systems, such as TaiChi, Liuzijue, Wuqinxi, Baduanjin, Yijinjing, and Yoga, have gained increasing attention. These practices integrate breath regulation, mental focus, and gentle physical movements, offering advantages of simplicity, safety, and sustainability, and are considered valuable adjuncts to conventional pulmonary rehabilitation for chronic lung diseases (9). Existing studies have demonstrated that such traditional mind–body exercises can improve pulmonary function, exercise capacity, and quality of life in COPD patients (10–14). However, systematic comparisons of the differential therapeutic effects among these exercise modalities remain limited. Therefore, this study aimed to compare the clinical effects of various traditional mind–body exercise interventions on patients with stable COPD through a network meta-analysis, rank their relative efficacy, and provide evidence-based recommendations for the development of individualized rehabilitation strategies.

## 2 Materials and methods

### 2.1 Protocol

This systematic review and network meta-analysis was registered with the International Prospective Register of Systematic Reviews (PROSPERO; registration number CRD420251051806). The study was conducted in accordance with the Preferred Reporting Items for Systematic Reviews and Meta-Analyses for Network Meta-Analyses (PRISMA-NMA) (15).

### 2.2 Search strategy

A comprehensive literature search was performed across English-language databases (PubMed, Embase, Web of Science, and Cochrane Library) and Chinese databases (CNKI, Wanfang, and VIP) from their inception to June 2025. The search combined Medical Subject Headings (MeSH) and free-text terms, including “Qigong,” “chronic obstructive pulmonary disease,” “Mind-Body Therapy,” “TaiChi,” “Baduanjin,” “Yijinjing,” “Wuqinxi,” “Liuzijue,” and “Yoga.” Detailed search strategies for each database are provided in Supplementary Table 1.

### 2.3 Inclusion criteria

Studies were included based on the PICOS framework as follows: (1) Participants: Patients diagnosed with stable COPD according to the Global Initiative for Chronic Obstructive Lung Disease (GOLD) guidelines (2), aged ≥18 years, with no sex restriction; (2) Intervention: Single traditional mind–body exercise interventions, including TaiChi, Wuqinxi, Liuzijue, Baduanjin, Yijinjing, or Yoga; (3) The control group received conventional care, no intervention, or one of the six traditional mind–body exercises; (4) Outcomes: Percentage of predicted forced expiratory volume in 1 s (FEV1%), 6-min walk test (6MWT), and COPD Assessment Test (CAT) score; (5) Study type: Randomized controlled trials (RCTs).

### 2.4 Exclusion criteria

Studies were excluded if they met any of the following criteria: (1) Case reports, experimental protocols, or animal studies; (2) Review articles, conference abstracts, or theses; (3) Studies with incomplete data or non-extractable outcomes; (4) Duplicate publications.

### 2.5 Study selection and data extraction

All retrieved references were imported into Endnote X9, where duplicates were removed. Two investigators independently screened titles and abstracts based on the inclusion and exclusion criteria, followed by a full-text review for potentially eligible studies. Data extraction was also performed independently by two investigators, with discrepancies resolved by a third investigator through discussion until consensus was reached. Extracted data included the first author’s name, publication year, country, sample size, participant age, intervention frequency and duration, and study outcomes. All extracted information was double-checked for accuracy, and any inconsistencies were resolved through group discussion. Where standard deviations (SDs) were not provided, they were derived from standard errors (SEs), confidence intervals (CIs), or t or *p*-values, or attempts were made to contact the authors at least three times through e-mails to collect the missing data.

### 2.6 Assessment of risk of bias

The Cochrane Collaboration’s Risk of Bias tool was used to assess the quality of included RCTs (16). The following domains were evaluated: sequence generation, allocation concealment, blinding of participants and personnel, blinding of outcome assessors, completeness of outcome data, selective outcome reporting, and other potential sources of bias. Each domain was rated as “low risk,” “high risk,” or “unclear risk.” Assessments were performed independently by two investigators, with disagreements resolved by a third reviewer through discussion.

### 2.7 Statistical analysis

All analyses were performed using Stata version 14.0. For continuous outcomes (FEV1%, 6MWT, and CAT), post-intervention means and standard deviations were analyzed. Since all studies used consistent scales, mean difference (MD) and 95% confidence intervals (CIs) were calculated as effect estimates. A multivariate random-effects meta-analysis was performed using the “Network” package, which accounts for heterogeneity caused by clinical and other factors across studies and provides a more conservative confidence interval for pooled point estimates. A network plot was generated to show the comparative relationships among the groups. Network transitivity was assessed by comparing clinical and methodological characteristics to ensure that multiple treatment comparisons were sufficiently similar. Probability values were summarized and reported as the surface under the cumulative ranking (SUCRA) curve. The SUCRA value would be 0 when a treatment is most likely the worst and 1 when it is most likely the best. Publication bias and small-study effects were detected through an asymmetrical funnel plot.

## 3 Results

### 3.1 Selection process

**FIGURE 1:**
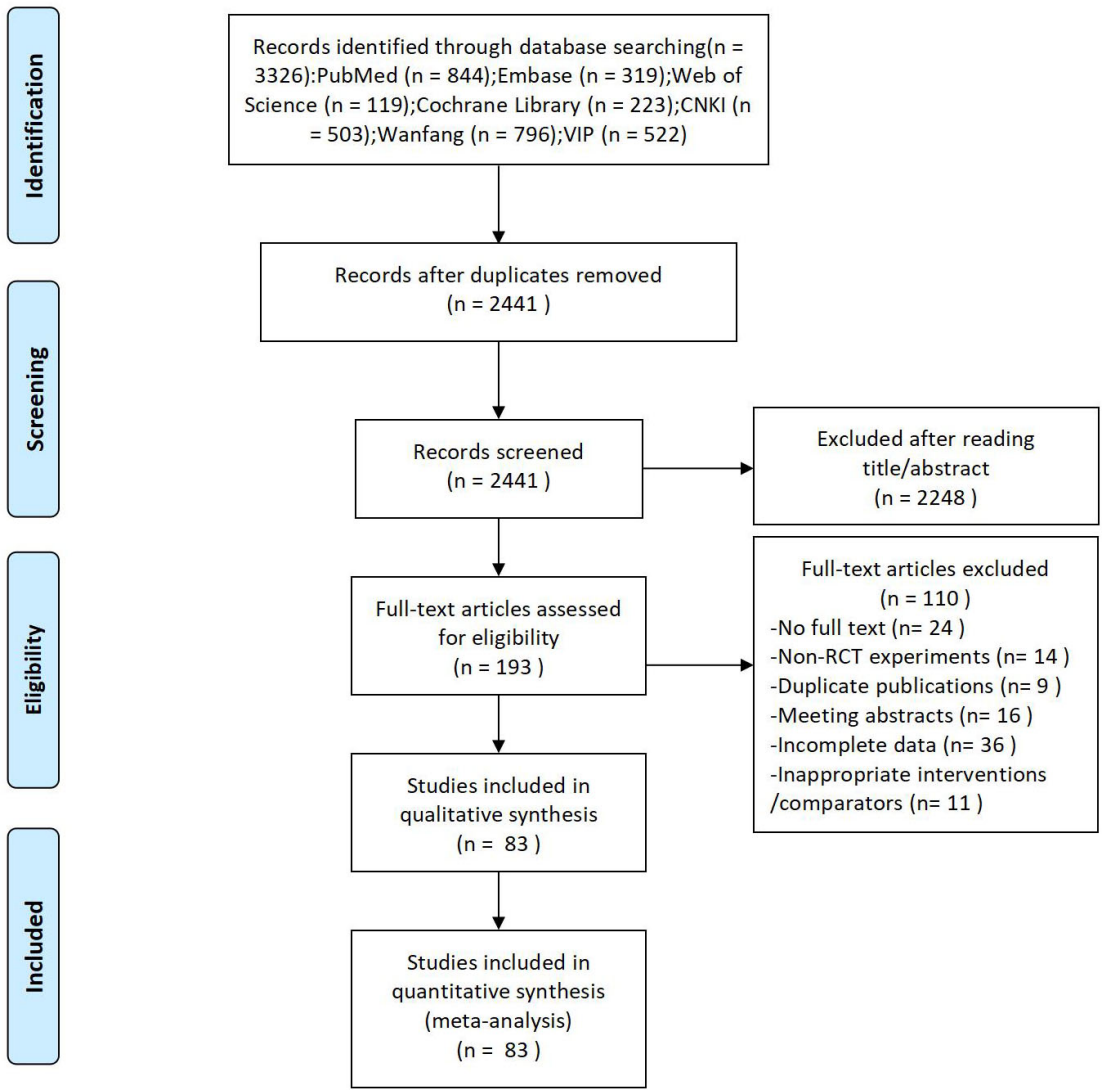
PRISMA flow diagram of the study selection process.

According to the predefined search strategy, a total of 3,326 records were initially identified. After removing duplicates and screening titles and abstracts, 193 articles remained. Following full-text assessment based on the inclusion and exclusion criteria, 83 RCTs were ultimately included in the network meta-analysis. The study selection process and details of the included studies are presented in Figure 1.

### 3.2 Characteristics of included studies

A total of 83 studies were included, encompassing 6,707 patients for the network meta-analysis, with 3,355 in the intervention groups and 3,352 in the control groups. Among these studies, 15 investigated TaiChi, 21 examined Liuzijue, 6 assessed Wuqinxi, 32 evaluated Baduanjin, 3 explored Yijinjing, and 6 involved Yoga. Full bibliographic details of the included studies are provided in Supplementary Table 2.

### 3.3 Risk of bias assessment

**FIGURE 2:**
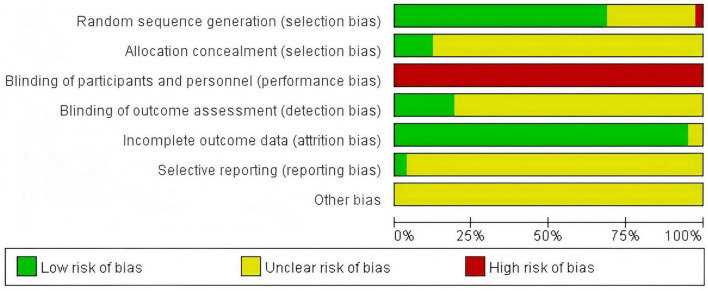
Risk of bias graph.

The assessment of risk of bias is presented in Figure 2. Among the 83 included RCTs, 57 studies clearly described methods for random sequence generation, whereas most did not provide detailed information on allocation concealment, with only 10 studies reporting specific concealment procedures. Due to the inherent nature of the interventions, blinding of participants and personnel was generally not feasible, and only 16 studies implemented blinding of outcome assessors. Overall, most studies demonstrated adequate data completeness, but the risks of selective reporting and other potential sources of bias were frequently rated as “unclear.” Detailed assessments of risk of bias for each study are available in Supplementary Figure 1.

### 3.4 Network meta-analysis

**FIGURE 3:**
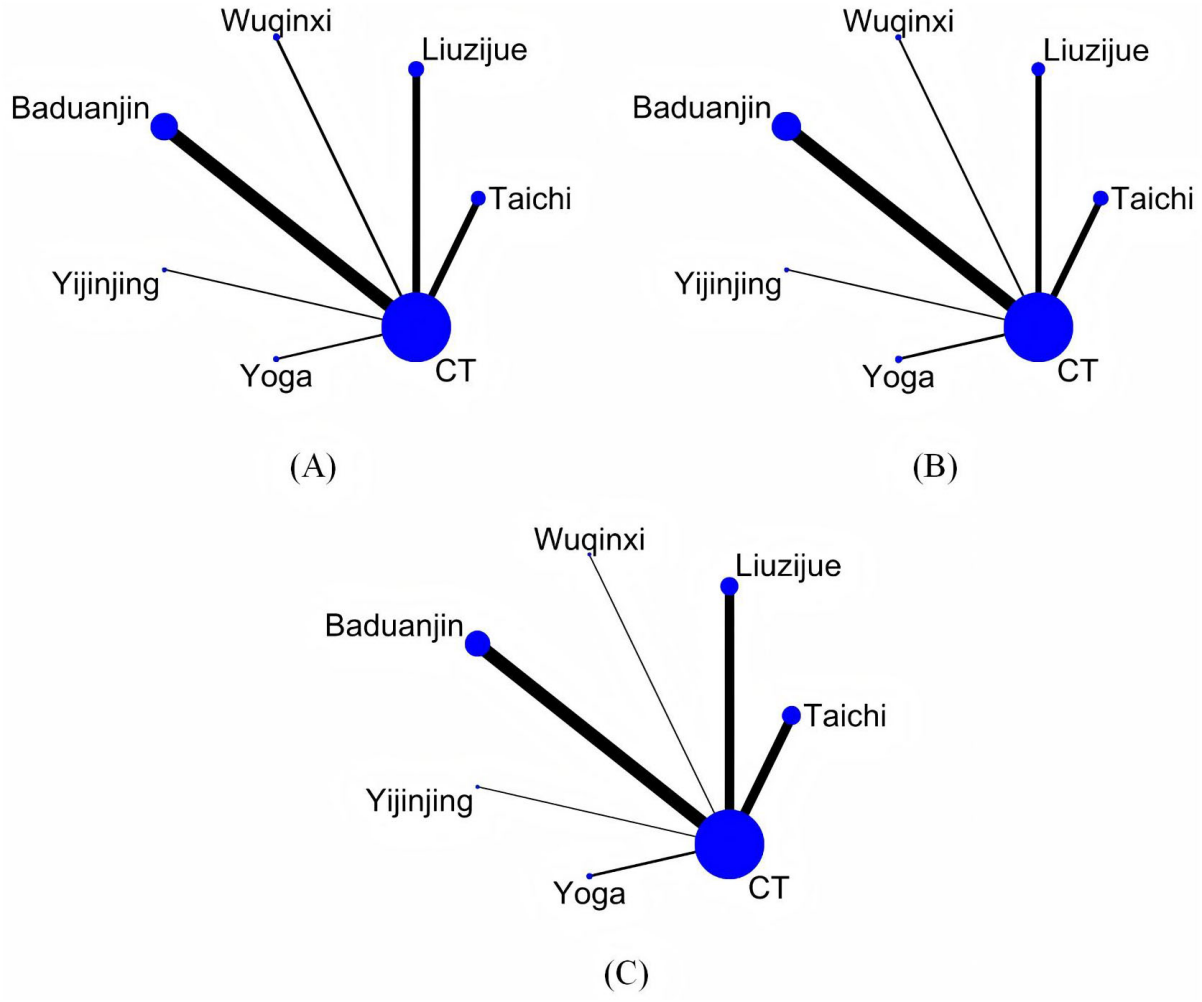
Network evidence plot. **(A)** FEV1%, **(B)** 6MWT, **(C)** CAT. CT conventional treatment.

Figures 3A–C illustrate the network structures constructed for the three outcome measures–FEV1%, 6MWT and CAT score–across the included traditional mind–body exercise interventions in patients with COPD. Each node represents a specific intervention, with the node size proportional to the total sample size of the studies evaluating that intervention. Lines connecting two nodes indicate the presence of direct head-to-head randomized controlled trials comparing those interventions. The absence of a connecting line indicates that no direct comparison exists between the two interventions, and the network therefore did not form any closed loops. Nevertheless, indirect comparisons could still be made through the shared control group. Accordingly, inconsistency testing was not required in this study.

**FIGURE 4:**
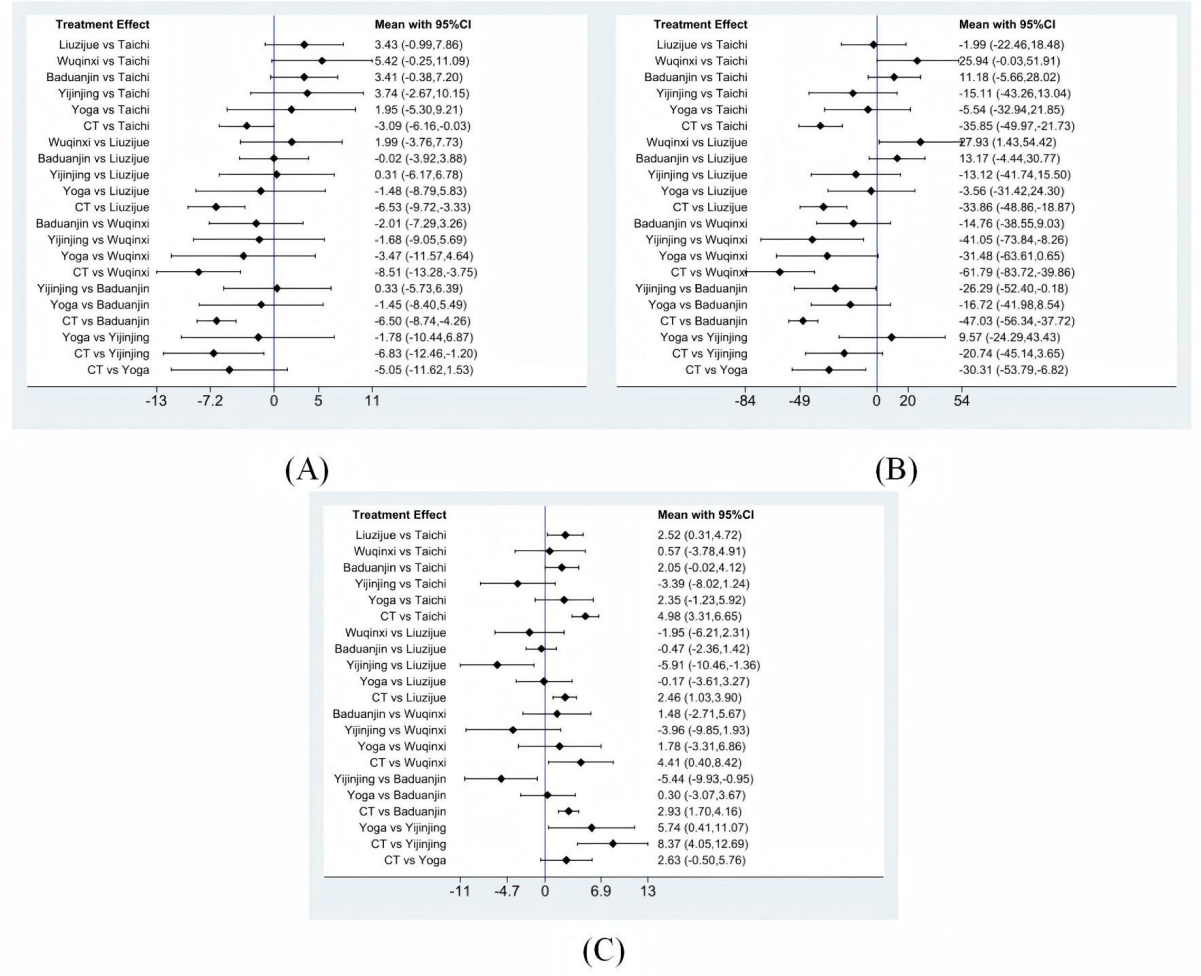
Forest plots. **(A)** FEV1%, **(B)** 6MWT, **(C)** CAT. CT conventional treatment.

For FEV1%, 60 studies comprising 4,465 patients were included. Compared with conventional treatment, significant improvements in pulmonary function were observed with TaiChi (MD = 3.09, 95% CI 0.03 to 6.16), Liuzijue (MD = 6.53, 95% CI 3.33 to 9.72), Wuqinxi (MD = 8.51, 95% CI 3.75 to 13.28), Baduanjin (MD = 6.50, 95% CI 4.26 to 8.74), and Yijinjing (MD = 6.83, 95% CI 1.20 to 12.46). Details are presented in Figure 4A. Based on SUCRA probabilities, the ranking for improving FEV1% was: Wuqinxi (82.9%) > Yijinjing (65.9%) > Liuzijue (63.5%) > Baduanjin (63.1%) > Yoga (47.8%) > TaiChi (25.3%) > Conventional treatment (1.5%). Full rankings are provided in Table 1 and Supplementary Figure 2A.

**TABLE 1:** Ranking table of SUCRA values.

Intervention	FEV1%	6MWT	CAT
Taichi	25.3	53.1	75.9
Liuzijue	63.5	49.1	33.0
Wuqinxi	82.9	96.7	63.2
Baduanjin	63.1	80.6	42.4
Yijinjing	65.9	26.8	96.7
Yoga	47.8	42.9	37.7
CT	1.5	0.9	1.2

CT, conventional treatment.

For 6MWT, 59 studies including 4,517 patients were analyzed. Compared with conventional treatment, significant improvements in exercise capacity were found for TaiChi (MD = 35.85, 95% CI 21.73 to 49.97), Liuzijue (MD = 33.86, 95% CI 18.87 to 48.86), Wuqinxi (MD = 61.79, 95% CI 39.86 to 83.72), Baduanjin (MD = 47.03, 95% CI 37.72 to 56.34), and Yoga (MD = 30.31, 95% CI 6.82 to 53.79). Additionally, Wuqinxi was significantly superior to Liuzijue (MD = 27.93, 95% CI 1.43 to 54.42) and Yijinjing (MD = 41.05, 95% CI 8.26 to 73.84), and Baduanjin was significantly superior to Yijinjing (MD = 26.29, 95% CI 0.18 to 52.40). Figure 4B shows the results. SUCRA rankings for 6MWT improvements were: Wuqinxi (96.7%) > Baduanjin (80.6%) > TaiChi (53.1%) > Liuzijue (49.1%) > Yoga (42.9%) > Yijinjing (26.8%) > Conventional treatment (0.9%). Rankings are detailed in Table 1 and Supplementary Figure 2B.

For CAT scores, 28 studies with a total of 2,678 patients were included. Compared with conventional treatment, significant reductions in CAT scores were achieved by TaiChi (MD = −4.98, 95% CI −6.65 to −3.31), Liuzijue (MD = −2.46, 95% CI −3.90 to −1.03), Wuqinxi (MD = −4.41, 95% CI −8.42 to −0.40), Baduanjin (MD = −2.93, 95% CI −4.16 to −1.70), and Yijinjing (MD = −8.37, 95% CI −12.69 to −4.05). Furthermore, Yijinjing was significantly more effective than Liuzijue (MD = −5.91, 95% CI −10.46 to −1.36), Baduanjin (MD = −5.44, 95% CI −9.93 to −0.95), and Yoga (MD = −5.74, 95% CI −11.07 to −0.41). The results are presented in Figure 4C. Based on SUCRA values, the ranking for CAT reduction was: Yijinjing (96.7%) > TaiChi (75.9%) > Wuqinxi (63.2%) > Baduanjin (42.4%) > Yoga (37.7%) > Liuzijue (33.0%) > Conventional treatment (1.2%). Rankings are summarized in Table 1 and Supplementary Figure 2C.

### 3.5 Publication bias analysis

Funnel plots for FEV1%, 6MWT, and CAT outcomes were generated (Supplementary Figures 3A–C). Overall, the scatter points in all three funnel plots were approximately symmetrically distributed on both sides of the inverted funnel, with no substantial asymmetry observed. Except for a few studies located at the funnel edges, the distribution was relatively balanced, indicating no significant small-study effects or apparent publication bias.

## 4 Discussion

Chronic obstructive pulmonary disease is a common chronic respiratory condition with high disability and mortality rates, posing a substantial burden on global public health systems (17, 18). Although pharmacological treatments can effectively control symptoms and reduce the risk of acute exacerbations, they have limited impact on altering the natural course of COPD or halting the long-term decline in lung function (19). Consequently, comprehensive rehabilitation interventions centered on exercise training have become increasingly important as adjunctive strategies for the management of stable COPD (20). This study synthesized evidence from 83 randomized controlled trials and systematically evaluated the clinical efficacy of six traditional mind–body exercises–TaiChi, Liuzijue, Wuqinxi, Baduanjin, Yijinjing, and Yoga–on FEV1%, 6MWT, and CAT scores in patients with stable COPD. The analysis demonstrated that Wuqinxi ranked highest for improving pulmonary function and exercise capacity, while Yijinjing showed the most pronounced benefits in enhancing quality of life. These findings suggest that different mind–body exercise interventions may contribute to COPD rehabilitation through distinct physiological and psychological mechanisms, allowing clinicians to tailor interventions according to patients’ functional status and rehabilitation needs.

Declines in pulmonary function and exercise capacity in COPD patients result from a combination of pathological changes. Chronic airway inflammation and alveolar destruction lead to airflow limitation and lung hyperinflation, which increase residual lung volume, exacerbate respiratory mechanical loads, and reduce breathing efficiency (21). Additionally, skeletal muscle dysfunction is frequently observed, characterized by muscle fiber atrophy, mitochondrial impairment, and reduced capillary density, all of which compromise metabolic capacity and contribute to substantial losses in muscle strength and endurance (22). Chronic systemic inflammation and physical deconditioning due to prolonged inactivity further aggravate these impairments, creating a vicious cycle of dyspnea and exercise intolerance during daily activities. Wuqinxi imitates the movements and physical traits of five animals, namely the tiger, deer, bear, monkey, and bird, to cultivate strength, flexibility, steadiness, agility, and lightness, thereby promoting musculoskeletal relaxation and facilitating meridian flow. Gao et al. (23) showed that Wuqinxi helps improve pulmonary circulation, increase blood oxygen saturation, strengthen the inspiratory, expiratory, and accessory respiratory muscles, and enhance the regulation of the autonomic nervous system. This may be attributed to its incorporation of large-amplitude movements such as forward flexion, backward extension, lateral bending, and trunk rotation, combined with upper-limb stretching/chest expansion synchronized with deep breathing, thereby forming a rhythmic “movement–breath coordination.” These elements are expected to improve chest wall compliance and diaphragmatic excursion, enhance recruitment of inspiratory, expiratory, and accessory respiratory muscles, reduce ventilatory demand under a given workload, and alleviate dynamic hyperinflation, which may in turn translate into improvements in FEV1% and 6MWT performance. Previous studies have confirmed that Wuqinxi can improve pulmonary function and exercise capacity in COPD patients, and our network meta-analysis further demonstrates its superior overall ranking for enhancing FEV1% and 6MWT performance compared with other mind–body exercises (12).

Chronic obstructive pulmonary disease often results in impaired quality of life due to persistent symptoms and functional limitations. Quality of life not only reflects the overall burden of disease but also serves as a critical endpoint for evaluating disease severity and therapeutic efficacy (24). The CAT, with its simplicity, ease of use, and high clinical sensitivity, is widely employed for assessing quality of life in COPD patients (25). In this study, Yijinjing yielded the most significant improvements in CAT scores. Previous studies have shown (26) that Yijinjing can effectively improve pulmonary function, enhance self-efficacy in emotion regulation, and promote daily physical activity. Moreover, Yijinjing training can significantly increase the proportions of CD4^+^ and NK cells as well as the CD4^+^/CD8^+^ ratio, accompanied by elevated levels of IL-2 and TNF-α and decreased IL-6, suggesting potential immunoregulatory and anti-aging effects (27). These effects may, to some extent, explain its advantages in improving quality of life.

This study has several limitations. First, the literature search was limited to Chinese and English databases, which may have excluded relevant studies published in other languages and thus affected the comprehensiveness of the evidence base. Second, although most trials mentioned randomization, only a minority explicitly reported allocation concealment. Due to the nature of the interventions, blinding of participants and personnel was generally not feasible, which may have introduced performance bias. Third, substantial heterogeneity existed among the included studies in terms of study design, intervention formats, delivery methods, and duration, with no standardized protocols, potentially complicating comparisons. Small sample sizes in some studies may also have exaggerated intervention effects. Fourth, because the evidence network in this study did not form closed loops and many comparisons were based mainly on indirect evidence, SUCRA, as a probability-based ranking metric, is inherently sensitive to uncertainty. Therefore, the ranking results should be interpreted with caution and considered together with the effect sizes and their 95% confidence intervals. Fifth, compared with Baduanjin and TaiChi, the number of RCTs investigating Liuzijue, Wuqinxi, Yijinjing, and Yoga was relatively limited, which may affect the stability and generalizability of their efficacy estimates. Future high-quality, multi-arm RCTs are warranted to systematically evaluate the impact of traditional mind–body exercises on pulmonary function, exercise capacity, quality of life, and biological markers (e.g., inflammatory mediators, immune factors) in COPD patients, providing robust evidence for the optimization and clinical application of exercise-based rehabilitation strategies.

## 5 Conclusion

Wuqinxi demonstrated the most substantial improvements in pulmonary function and exercise capacity, whereas Yijinjing provided the greatest benefits in enhancing quality of life among patients with stable COPD. Different mind–body exercise interventions may facilitate rehabilitation through distinct physiological and psychological mechanisms, allowing clinicians to individualize intervention selection according to patients’ functional status and rehabilitation goals.

## Data Availability

The original contributions presented in this study are included in this article/Supplementary material, further inquiries can be directed to the corresponding author.
