# Supplementary material for: Comparative efficacy of different traditional mind-body exercises in patients with stable chronic obstructive pulmonary disease: a systematic review and network meta-analysis
Source: Front Med (Lausanne). 2025 Oct 10;12:1678352. doi: 10.3389/fmed.2025.1678352 (PMC12549297; doi:10.3389/fmed.2025.1678352)
Supplement: Supplementary file 1 [file Data_Sheet_1.docx]

**Supplementary Material**

**Table S1.** Detailed searching strategies

(1) Search strategy in PubMed（n = 844）

| #1 | (((((((((("Pulmonary Disease, Chronic Obstructive"[Mesh]) OR (Chronic Obstructive Pulmonary Diseases[Title/Abstract])) OR (COPD[Title/Abstract])) OR (Chronic Obstructive Lung Disease[Title/Abstract])) OR (Chronic Obstructive Pulmonary Disease[Title/Abstract])) OR (COAD[Title/Abstract])) OR (Chronic Obstructive Airway Disease[Title/Abstract])) OR (Airflow Obstruction, Chronic[Title/Abstract])) OR (Airflow Obstructions, Chronic[Title/Abstract])) OR (Chronic Airflow Obstructions[Title/Abstract])) OR (Chronic Airflow Obstruction[Title/Abstract]) |
| --- | --- |
| #2 | (((((("Mind-Body Therapies"[Mesh]) OR (Mind Body Therapies[Title/Abstract])) OR (Mind-Body Therapy[Title/Abstract])) OR (Therapies, Mind-Body[Title/Abstract])) OR (Therapy, Mind-Body[Title/Abstract])) OR (Mind-Body Medicine[Title/Abstract])) OR (Mind Body Medicine[Title/Abstract]) |
| #3 | (((((((Yoga[Title/Abstract]) OR (Tai chi[Title/Abstract])) OR (Taiji[Title/Abstract])) OR (Baduanjin[Title/Abstract])) OR (Yijinjing[Title/Abstract])) OR (Wuqinxi[Title/Abstract])) OR (Liuzijue[Title/Abstract])) OR (Qigong[Title/Abstract]) |
| #4 | #1 AND #2 AND #3 |

(2) Search strategy in Embase（n = 319）

| #1 | 'pulmonary disease, chronic obstructive':ti,ab,kw OR 'chronic obstructive pulmonary diseases':ti,ab,kw OR copd:ti,ab,kw OR 'chronic obstructive lung disease':ti,ab,kw OR 'chronic obstructive pulmonary disease':ti,ab,kw OR coad:ti,ab,kw OR 'chronic obstructive airway disease':ti,ab,kw OR 'airflow obstruction, chronic':ti,ab,kw OR 'airflow obstructions, chronic':ti,ab,kw OR 'chronic airflow obstructions':ti,ab,kw OR 'chronic airflow obstruction':ti,ab,kw |
| --- | --- |
| #2 | 'mind-body therapies':ti,ab,kw OR 'mind body therapies':ti,ab,kw OR 'mind-body therapy':ti,ab,kw OR 'therapies, mind-body':ti,ab,kw OR 'therapy, mind-body':ti,ab,kw OR 'mind-body medicine':ti,ab,kw OR 'mind body medicine':ti,ab,kw |
| #3 | yoga:ti,ab,kw OR 'tai chi':ti,ab,kw OR taiji:ti,ab,kw OR baduanjin:ti,ab,kw OR yijinjing:ti,ab,kw OR wuqinxi:ti,ab,kw OR liuzijue:ti,ab,kw OR qigong:ti,ab,kw |
| #4 | #1 AND #2 AND #3 |

(3) Search strategy in Web of science（n = 119）

| #1 | ((((((((((TS=(Pulmonary Disease, Chronic Obstructive)) OR TS=(Chronic Obstructive Pulmonary Diseases)) OR TS=(COPD)) OR TS=(Chronic Obstructive Lung Disease)) OR TS=(Chronic Obstructive Pulmonary Disease)) OR TS=(COAD)) OR TS=(Chronic Obstructive Airway Disease)) OR TS=(Airflow Obstruction, Chronic)) OR TS=(Airflow Obstructions, Chronic)) OR TS=(Chronic Airflow Obstructions)) OR TS=(Chronic Airflow Obstruction) |
| --- | --- |
| #2 | ((((((TS=(Mind-Body Therapies)) OR TS=(Mind Body Therapies)) OR TS=(Mind-Body Therapy)) OR TS=(Therapies, Mind-Body)) OR TS=(Therapy, Mind-Body)) OR TS=(Mind-Body Medicine)) OR TS=(Mind Body Medicine) |
| #3 | (((((((TS=(Yoga)) OR TS=(Tai chi)) OR TS=(Taiji)) OR TS=(Baduanjin)) AND TS=(Yijinjing)) AND TS=(Wuqinxi)) AND TS=(Liuzijue)) AND TS=(Qigong) |
| #4 | #1 AND #2 AND #3 |

(4) Search strategy in Cochrane Library（n = 223）

| #1 | (Pulmonary Disease, Chronic Obstructive):ti,ab,kw OR (Chronic Obstructive Pulmonary Diseases):ti,ab,kw OR (COPD):ti,ab,kw OR (Chronic Obstructive Lung Disease):ti,ab,kw OR (Chronic Obstructive Pulmonary Disease):ti,ab,kw |
| --- | --- |
| #2 | (COAD):ti,ab,kw OR (Chronic Obstructive Airway Disease):ti,ab,kw OR (Airflow Obstruction, Chronic):ti,ab,kw OR (Airflow Obstructions, Chronic):ti,ab,kw OR (Chronic Airflow Obstructions):ti,ab,kw |
| #3 | (Chronic Airflow Obstruction):ti,ab,kw |
| #4 | #1 OR #2 OR #3 |
| #5 | (Mind-Body Therapies):ti,ab,kw OR (Mind Body Therapies):ti,ab,kw OR (Mind-Body Therapy):ti,ab,kw OR (Therapies, Mind-Body):ti,ab,kw OR (Therapy, Mind-Body):ti,ab,kw |
| #6 | (Mind-Body Medicine):ti,ab,kw OR (Mind Body Medicine):ti,ab,kw |
| #7 | #5 OR #6 |
| #8 | (Yoga):ti,ab,kw OR (Tai chi):ti,ab,kw OR (Taiji):ti,ab,kw OR (Baduanjin):ti,ab,kw OR (Yijinjing):ti,ab,kw |
| #9 | (Wuqinxi):ti,ab,kw OR (Liuzijue):ti,ab,kw OR (Qigong):ti,ab,kw |
| #10 | #8 OR #9 |
| #11 | #4 OR #7 OR #10 |

(5) Search strategy in CNKI（n = 503）

| #1 | 主题=（慢性阻塞性肺疾病 + 慢阻肺 + COPD） AND 主题=（身心运动 + 身心锻炼 + 气功 + 瑜伽 + 太极拳 + 八段锦 + 易筋经 + 五禽戏 + 六字诀） |
| --- | --- |

(6) Search strategy in Wanfang（n = 796）

| #1 | 主题=（慢性阻塞性肺疾病 OR 慢阻肺 OR COPD） AND 主题=（身心运动 OR 身心锻炼 OR 气功 OR 瑜伽 OR 太极拳 OR 八段锦 OR 易筋经 OR 五禽戏 OR 六字诀） |
| --- | --- |

(7) Search strategy in VIP（n = 796）

| #1 | 主题=（慢性阻塞性肺疾病 OR 慢阻肺 OR COPD） AND 主题=（身心运动 OR 身心锻炼 OR 气功 OR 瑜伽 OR 太极拳 OR 八段锦 OR 易筋经 OR 五禽戏 OR 六字诀） |
| --- | --- |

**Table S2. The basic characteristics of included studies**

| Study | Country | Sample size  (EG/CG) | Age  (years, EG/CG) | Interventions  (EG/CG) | frequency and duration | outcomes |
| --- | --- | --- | --- | --- | --- | --- |
| Chan 2011^[1]^ | China | 137(70/67) | 71.7 ± 8.2/73.6 ± 7.4 | Taichi/CT | 60 min/time, 2 times/week,3 months | ② |
| Du 2013^[2]^ | China | 74(36/38) | 65.24 + 8.37/64.48 + 6.54 | Taichi/CT | 30 min/time, 2 times/day, 12 weeks | ①②③ |
| Hu 2020^[3]^ | China | 84(42/42) | 62.09 + 3.80/62.20 + 3.51 | Taichi/CT | 35 min/time, 2 times/day, 3 months | ① |
| Kantatong 2020^[4]^ | Thailand | 50(25/25) | 69.68 ± 7.67/67.48 ± 10.17 | Taichi/CT | 2 times/week, 12 weeks + 3 times/week, 12 weeks | ② |
| Li 2016^[5]^ | China | 40(20/20) | 60.3 ± 6.9 | Taichi/CT | 40 min/time, 3 times/week, 3 months | ①② |
| Liu 2019^[6]^ | China | 100(50/50) | 53.5 ± 6.1/53.1 ± 5.6 | Taichi/CT | 30 min/time, 2 times/day, 12 months | ①②③ |
| Niu 2014^[7]^ | China | 40(20/20) | 59.7 ± 2.76/61.3 + 2.89 | Taichi/CT | 50 min/time, once/day, 6 months | ①② |
| Pan 2018^[8]^ | China | 41(20/21) | NA | Taichi/CT | 30 min/time, 3 times/week, 8 weeks | ①②③ |
| Peng 2020^[9]^ | China | 80(40/40) | NA | Taichi/CT | 30 min/time, once/day, 24 weeks | ②③ |
| Wang 2019^[10]^ | China | 50(26/24) | 67.83 ± 5.32/67.86 ± 5.98 | Taichi/CT | 60 min/time, 3 times/week, 3 months | ①②③ |
| Yao 2004^[11]^ | China | 80(40/40) | 66.1 ± 4/66.2 ± 4.2 | Taichi/CT | 30 min/time, once/day, 3 months | ① |
| Zhang(1) 2019^[12]^ | China | 58(29/29) | 49.24 + 2.19/50.13 + 2.08 | Taichi/CT | 60 min/time, 2 times/day, 24 months | ① |
| Zhu 2018^[13]^ | China | 60(30/30) | 67.87 ± 5.22/68.10 ± 6.57 | Taichi/CT | 40-50 min/time, 3 times/week, 3 months | ①②③ |
| Zhang 2012^[14]^ | China | 60(30/30) | 62.00 ± 7.30/62.34 ± 6.88 | Taichi/CT | once/day, 12 months | ①② |
| Zhang 2014^[15]^ | China | 36(18/18) | 68.02 ± 6.91/66.71 ± 5.84 | Taichi/CT | 60 min/time, 2 times/day, 12 months | ①②③ |
| Chen 2008^[16]^ | China | 40(21/19) | 71.76 ± 7.31/73.32 ± 6.33 | Liuzijue/CT | 3 months | ① |
| Chen 2009^[17]^ | China | 60(31/29) | 70.16 ± 9.45/71.52 ± 9.67 | Liuzijue/CT | 30 min/time, 1 times/day, 3 months | ①② |
| Chen(1) 2024^[18]^ | China | 64(32/32) | 63.68 ± 12.86/63.89 ± 13.02 | Liuzijue/CT | 30 min/time, 2 times/day, 3 months | ③ |
| Deng 2018^[19]^ | China | 60(30/30) | 72.37 ± 5.95/72.60 ± 8.24 | Liuzijue/CT | 30 min/time, once/day, 5 times/week, 3 months | ② |
| Deng(1) 2020^[20]^ | China | 62(30/32) | 76.53 ± 8.59/76.59 ± 7.69 | Liuzijue/CT | 30 min/time, once/day, 3 months | ①③ |
| Fang 2012^[21]^ | China | 121(61/60) | 71.75 ± 9.38/73.10 ± 12.39 | Liuzijue/CT | 30 min/time, once/day, 6 months | ② |
| Fang 2022^[22]^ | China | 60(30/30) | 68.72 ± 5.43/68.23 ± 5.27 | Liuzijue/CT | 40 min/time, 5 times/week, 3 months | ①② |
| Hu 2021^[23]^ | China | 40(20/20) | NA | Liuzijue/CT | 30 min/time, 3 times/day, 6 months | ①②③ |
| Ji 2019^[24]^ | China | 57(28/29) | 63.75 ± 5.48/64.52 ± 5.68 | Liuzijue/CT | 30 min/time, 5 times/day, 3 months | ① |
| Lan 2016^[25]^ | China | 84(42/42) | 67.24 + 3.21/67.02 + 3.48 | Liuzijue/CT | 60 min/time, 2 times/day, 5 days/week, 12 weeks | ①③ |
| Li 2018^[26]^ | China | 36(17/19) | 66 ± 9/66 ± 9 | Liuzijue/CT | 60 min/time, 6 times/week, 6 months | ①② |
| Liu(1) 2021^[27]^ | China | 31(15/16) | 65 ± 8/66 ± 8 | Liuzijue/CT | 60 min/time, 2 times/week, 12 weeks | ② |
| Lu 2021^[28]^ | China | 274(135/139) | NA | Liuzijue/CT | 20 min/time, once/day, 12 weeks | ③ |
| Shen 2017^[29]^ | China | 100(50/50) | 72.15 ± 2.08/72.55 ± 2.15 | Liuzijue/CT | 20 min/time, once/day, 6 months | ①③ |
| Shi 2020^[30]^ | China | 60(30/30) | 64.34 ± 5.32/65.24 ± 5.22 | Liuzijue/CT | 30 min/time, once/day, 3 months | ③ |
| Sun 2019^[31]^ | China | 112(56/56) | 65.45 ± 6.24/64.78 ± 6.12 | Liuzijue/CT | 10 min/time, 2 times/day, 6 months | ①② |
| Wu(1) 2018^[32]^ | China | 33(16/17) | 67 ± 8/66 ± 9 | Liuzijue/CT | 40 min/time, 6 times/week, 6 months | ①② |
| Wu(2) 2018^[33]^ | China | 31(15/16) | 65 ± 8/66 ± 8 | Liuzijue/CT | 60 min/time, 2 times/week, 3 months | ① |
| Xiao 2015^[34]^ | China | 126(63/63) | 72.2 ± 1.7/70.9 ± 1.4 | Liuzijue/CT | 45 min/time, 4 times/week, 6 months | ② |
| Zhang(2) 2019^[35]^ | China | 120(60/60) | 71.30 ± 2.96/72.90 ± 3.25 | Liuzijue/CT | 30 min/time, once/day, 3 months | ② |
| Zhu 2011^[36]^ | China | 42(20/22) | NA | Liuzijue/CT | once/day, 3 months | ① |
| Cheng 2015^[37]^ | China | 93(48/45) | 58.66 ± 7.56/58.64 ± 7.52 | Wuqinxi/CT | 30 min/time, 1-2 times/day, ≥4 times/week, 6 months | ③ |
| Gao 2017^[38]^ | China | 72(36/36) | 67.14 ± 9.08/66.03 ± 8.18 | Wuqinxi/CT | 30 min/time, 2 times/day, 3 months | ①② |
| Liu 2020^[39]^ | China | 100(50/50) | 74.24 ± 9.10/67.72 ± 9.26 | Wuqinxi/CT | 45 min/time, 1 times/day, 3 months | ①② |
| Wei 2015^[40]^ | China | 93(48/45) | 58.66 ± 7.56/58.64 ± 7.52 | Wuqinxi/CT | 30 min/time, 1-2 times/day, ≥4 times/week, 6 months | ① |
| Zhao 2015^[41]^ | China | 60(30/30) | 58.91 ± 5.86/56.66 ± 6.43 | Wuqinxi/CT | 40 min/time, 3 times/day, 3 months | ①② |
| Zhu 2010^[42]^ | China | 47(26/21) | 53.53 ± 10.05/55.46 ± 9.87 | Wuqinxi/CT | 45 min/time, once/day, 3 months | ①② |
| Chen 2015^[43]^ | China | 61(31/30） | 66.26 ± 5.13/66.90 ± 4.63 | Baduanjin/CT | 30 min/time, once/day, 3 months | ②③ |
| Chen 2025^[44]^ | China | 108(55/53） | 66.7 ± 6.7/66.7 ± 8.1 | Baduanjin/CT | 30 min/time, 2 times/day, 5 days/week, 12 weeks, | ①②③ |
| Chen(2) 2024^[45]^ | China | 318(158/160） | 61.52 ± 10.31/61.97 ± 10.91 | Baduanjin/CT | 30 min/time, 2 times/day, ≥5 days/week, 6 months | ② |
| Deng 2015^[46]^ | China | 64(32/32) | 66.26 ± 5.13/66.90 ± 4.63 | Baduanjin/CT | 30 min/time, once/day, 3 months | ① |
| Deng(2) 2020^[47]^ | China | 54(27/27) | 64.84 ± 9.03/63.92 ± 8.47 | Baduanjin/CT | 30 min/time, once/day, ≥4 times/week, 6 months | ①② |
| Dong 2018^[48]^ | China | 92(46/46) | 63.97 ± 5.57/64.25 ± 6.01 | Baduanjin/CT | 30 min/time, once/day, ≥4 times/week, 6 months | ①②③ |
| Feng 2009^[49]^ | China | 60(30/30) | 62 ± 5/63 ± 4 | Baduanjin/CT | 45 min/time, 2 times/day, 24 weeks | ①② |
| Feng 2024^[50]^ | China | 84(42/42) | 53.01 ± 3.20/52.36 ± 3.14 | Baduanjin/CT | 40 min/time, once/day, 6 weeks | ②③ |
| Guo(1) 2016^[51]^ | China | 60(30/30) | 62.8/63.4 | Baduanjin/CT | 15-20 min/time, once/day, ≥4 times/week, 6 months | ① |
| Guo(2) 2016^[52]^ | China | 111(55/56) | 70.87 ± 6.07/70.79 ± 6.06 | Baduanjin/CT | 30 min/time, 4 times/week, 36 weeks | ①② |
| Guo(3) 2016^[53]^ | China | 320(161/159) | 64.15 ± 8.97/64.87 ± 8.86 | Baduanjin/CT | 30 min/time, once/day, ≥4 times/week, 6 months | ①③ |
| Huang 2017^[54]^ | China | 62(31/31) | 68.24 ± 3.28/69.77 ± 4.42 | Baduanjin/CT | 30 min/time, once/day, 6 months | ①③ |
| Jiang 2023^[55]^ | China | 80(40/40) | 57.28 ± 6.43/57.45 ± 6.39 | Baduanjin/CT | 25 min/time, once/day, 12 weeks | ②③ |
| Lei 2020^[56]^ | China | 99(49/50) | 60.45 ± 4.76/61.78 ± 5.32 | Baduanjin/CT | 60 min/time, once/day, 12 months | ② |
| Liang 2016^[57]^ | China | 82(41/41) | NA | Baduanjin/CT | 30 min/time, once/day, 3 months | ① |
| Liu 2013^[58]^ | China | 80(40/40) | 59.77 ± 7.08/60.67 ± 6.95 | Baduanjin/CT | 30 min/time, once/day, 3 months | ①② |
| Liu(2) 2021^[59]^ | China | 70(35/35) | 59.85 ± 15.83/60.25 ± 16.35 | Baduanjin/CT | 30 min/time, once/day, 4 weeks | ②③ |
| Pan 2016^[60]^ | China | 84(42/42) | 60.7 ± 5.6/61.8 ± 7.2 | Baduanjin/CT | 30 min/time, once/day, 6 months | ①② |
| Wang 2018^[61]^ | China | 76(38/38) | 63.17 ± 9.95/63.67 ± 9.75 | Baduanjin/CT | 30 min/time, once/day, 3 months | ①② |
| Wu 2019^[62]^ | China | 100(50/50) | 62.4 ± 5.6/60.7 ± 6.3 | Baduanjin/CT | 30 min/time, once/day, 3 months | ② |
| Wu 2020^[63]^ | China | 68(34/34) | 63.7 ± 3.6/64.2 ± 3.8 | Baduanjin/CT | 30 min/time, once/day, ＞4 times/week, 6 months | ①② |
| Wu 2024^[64]^ | China | 71(34/37) | 68.56 ± 4.63/66.22 ± 6.69 | Baduanjin/CT | 60 min/time, 3 days/week, 12weeks | ② |
| Yang 2016^[65]^ | China | 116(58/58) | 63.9 ± 6.7/63.0 ± 6.9 | Baduanjin/CT | ≥30 min/time, ≥180 min/week, 3 months | ①② |
| Yang 2017^[66]^ | China | 90(45/45) | 60.56 ± 5.84/60.34 ± 5.56 | Baduanjin/CT | 30 min/time, once/day, 3 months | ① |
| Ye 2016^[67]^ | China | 80(40/40) | 65.36 ± 5.28/64.98 ± 5.55 | Baduanjin/CT | 40 min/time, 5 times/week, 4 weeks | ② |
| Yin 2024^[68]^ | China | 100(50/50) | 70.87 ± 6.07/70.79 ± 6.06 | Baduanjin/CT | 30 min/time, once/day, 12 months | ①② |
| Yu 2019^[69]^ | China | 82(41/41) | 62.3 ± 1.2/62.3 ± 1.5 | Baduanjin/CT | 30 min/time, once/day, 3 months | ① |
| Yu 2021^[70]^ | China | 98(49/49) | 62.15 ± 7.33/61.93 ± 7.25 | Baduanjin/CT | 24 min/time, ≥5 times/week, 6 months | ①②③ |
| Zhang 2017^[71]^ | China | 60(30/30) | 68.50 ± 9.18/68.03 ± 7.92 | Baduanjin/CT | 2 months | ①② |
| Zhang(3) 2019^[72]^ | China | 60(30/30) | 65.46 ± 6.74/64.82 ± 6.23 | Baduanjin/CT | 30 min/time, once/day, 6 months | ①② |
| Zhu 2014^[73]^ | China | 123(63/60) | 69.0 ± 8.7/68.0 ± 9.2 | Baduanjin/CT | 30 min/time, 2 times/day,, 6 months | ①② |
| Zhu 2017^[74]^ | China | 215(106/109) | 67.00 ± 8.70/68.00 ± 9.20 | Baduanjin/CT | 40 min/time, 2 times/day,, 6 months | ① |
| Gao 2016^[75]^ | China | 112(55/57) | 71.42 ± 10.45/74.24 ± 10.34 | Yijinjing/CT | 60 min/time, 2 times/day,, 6 months | ①② |
| Zhang(1) 2016^[76]^ | China | 45(20/25) | 61.77 ± 4.07/59.35 ± 5.27 | Yijinjing/CT | 60 min/time, 2 times/day, 6 months | ①② |
| Zhang(2) 2016^[77]^ | China | 87(42/45) | 64.77 ± 11.07/62.35 ± 9.27 | Yijinjing/CT | 60 min/time, once/day, 6 months | ①②③ |
| Donesky 2009^[78]^ | USA | 29(14/15) | 72.2 ± 6.5/67.7 ± 11.5 | Yoga/CT | 60 min/time, 2 times/week, 12 weeks | ①② |
| Gupta 2014^[79]^ | India | 50(25/25) | 52.5 ± 3.9/52 ± 4.1 | Yoga/CT | 30 min/time, 2 times/day, 3 months | ①②③ |
| Ranjita 2016^[80]^ | India | 72(36/36) | 53.69 ± 5.66/54.41 ± 5.40 | Yoga/CT | 90 min/time, 6 times/week, 12 weeks | ② |
| Kaminsky 2017^[81]^ | USA | 43(21/22) | 68 ± 7/68 ± 9 | Yoga/CT | 30 min/time, once/day, 12 weeks | ①②③ |
| Thokchom 2018^[82]^ | India | 41(21/20) | 57.8 ± 2.68/60.65 ± 1.84 | Yoga/CT | 50 min/time, ≥5 times/week, 12 weeks | ② |
| Yudawhati 2019^[83]^ | Indonesia | 30(15/15) | 64.40 ± 10.45/65.33 ± 8.12 | Yoga/CT | 30 min/time, 2 times/week, 12 weeks | ① |

EG = experimental group, CG = control group, CT = conventional treatment, NA = not available, ①FEV1%, ②6MWT, ③ CAT.

**Figure S1.** Result of the risk of bias assessment

**
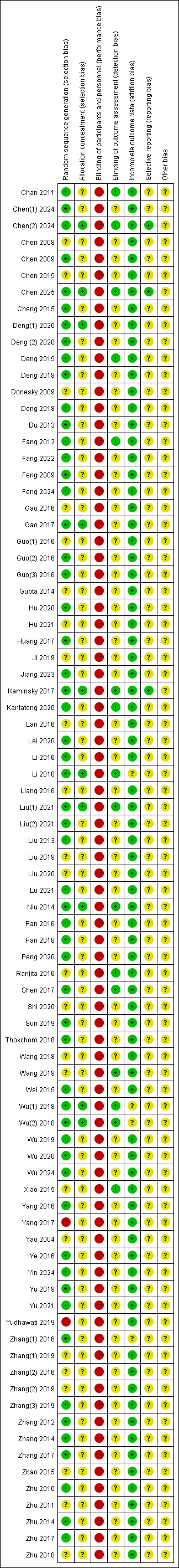
**

**Figure S2.** Cumulative ranking probability curves. (A) FEV1%, (B) 6MWT, (C) CAT.


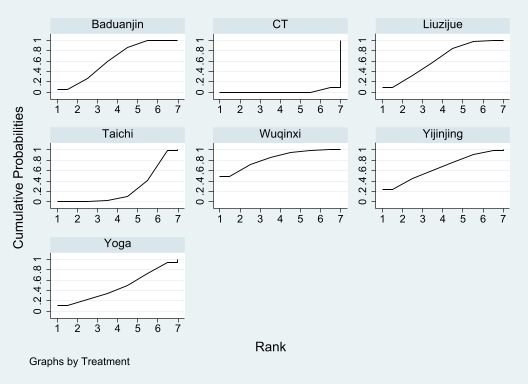


(A)


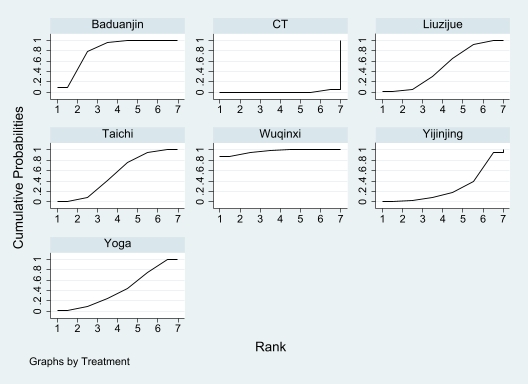


(B)


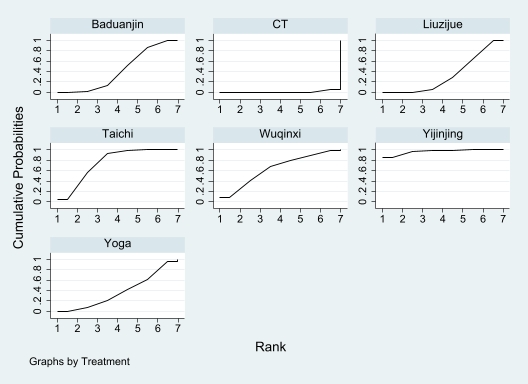


(C)

CT = conventional treatment.

**Figure S3.** Funnel plots. (A) FEV1%, (B) 6MWT, (C) CAT.


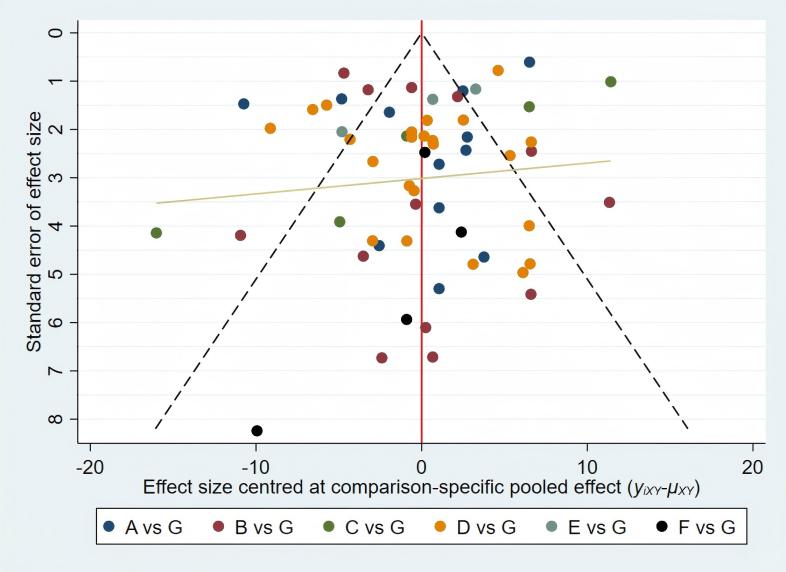


(A)


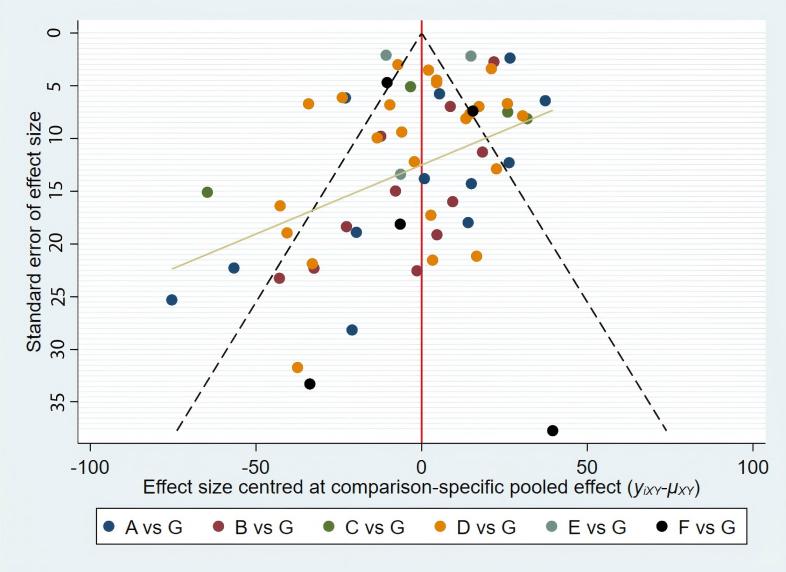


(B)


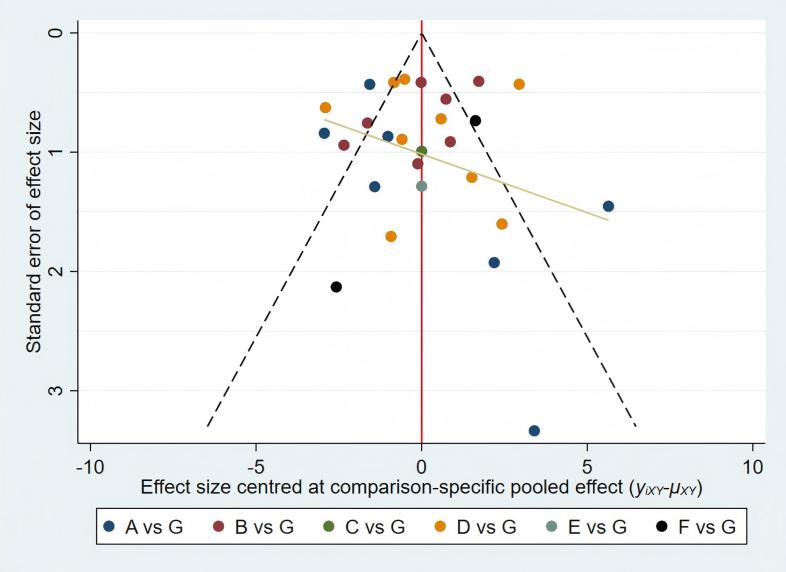


(C)

A = Taichi; B = Liuzijue; C = Wuqinxi; D = Baduanjin; E = Yijinjing; F = Yoga; G = conventional treatment.

# References

1. Chan AW, Lee A, Suen LKP, Tam WWS. Tai chi Qigong improves lung functions and activity tolerance in COPD clients: a single blind, randomized controlled trial. Complement Ther Med. (2011) 19:3-11. doi: 10.1016/j.ctim.2010.12.007.
2. Du S, Ding L, Wang C, Yang F, Xing B, Liu Z, et al. Effect of TaiChi exercise on exercise endurance and pulmonary function in patients with chronic obstructive pulmonary disease. Chin J Rehabil Med. (2013) 28:374-6.
3. Hu JP, Han PX, Sang XL, Hu JC, Wu XM, Mo L. Clinical efficacy of TaiChi rehabilitation training in middle-aged and elderly patients with stable chronic obstructive pulmonary disease. Chin J Geriatr. (2020) 40:5225-7.
4. Kantatong T, Panpanich R, Deesomchok A, Sungkarat S, Siviroj P. Effects of the tai chi qigong programme on functional capacity, and lung function in chronic obstructive pulmonary disease patients: A ramdomised controlled trial. J Tradit Complement Med. (2020) 10:354–9. doi: 10.1016/j.jtcme.2019.03.008.
5. Li SX, Li AJ, Sun J, Wang HX. Effect of Tai Chi rehabilitation on patients with chronic obstructive pulmonary disease. J Binzhou Med Univ. (2016) 39:275-7.
6. Liu DH, Zhang Y. Effect of TaiChi exercise on pulmonary function in middle-aged patients with stable chronic obstructive pulmonary disease. Chin Community Doctors. (2019) 35:154-7.
7. Niu R, He R, Luo BL, Hu C. The Effect of Tai Chi on Chronic Obstructive Pulmonary Disease: A Pilot Randomised Study of Lung Function, Exercise Capacity and Diaphragm Strength. Heart Lung Circ. (2014) 23:347–52. doi: 10.1016/j.hlc.2013.10.057.
8. Pan Y, Wang ZX, Min J, Xiao W. The effect of 24 simplified Taichi on pulmonary rehabilitation in patients with stable chronic obstructive pulmonary disease. Chin J Rehabil Med. (2018) 33:681-6.
9. Peng HY, Wang P, Qi CJ, Mao WA, Tao HH. Effect of six-movement Tai Chi rehabilitation form training on pulmonary rehabilitation of patients with chronic obstructive pulmonary disease in the stable stage. Chin Sci Technol J Database (Citation Edition) Med Health. (2020) 8:105-7.
10. Wang L, Wu K, Chen X, Liu Q. The effects of Tai Chi on lung function, exercise capacity and health related quality of life for patients with chronic obstructive pulmonary disease: a pilot study. Heart Lung Circ. (2019) 28:1206–12. doi: 10.1016/j.hlc.2018.05.204.
11. Yao YP. Observation on the rehabilitation effect of Tai Chi in patients with chronic obstructive pulmonary disease. Chin J Rehabil Theory Pract. (2004) 7:59-60.
12. Zhang Y, Liu DH. Effect of 24-form Tai Chi combined with respiratory rehabilitation training on pulmonary function and quality of life in patients with chronic obstructive pulmonary disease. J Pract Chin Med. (2019) 35:350-1.
13. Zhu S, Shi K, Yan J, He Z, Wang Y, Yi Q, et al. A modified 6-form Tai Chi for patients with COPD. Complementary Therapies in Medicine. (2018) 39:36–42. doi: 10.1016/j.ctim.2018.05.007.
14. Zhang LH, Wu JJ, Wang ZC. Effect of 24-form Tai Chi combined with respiratory rehabilitation training on pulmonary function and quality of life in patients with COPD. J Shanghai Univ Tradit Chin Med. (2012) 26:53-6.
15. Zhang XC, Cai YL, Zhang W, Chen XH, Jia XH, Zhang XY, et al. Effect of 24-form Tai Chi and respiratory function training combined with Western medicine on patients with stable chronic obstructive pulmonary disease. J Tradit Chin Med. (2014) 55:1937-41.
16. Chen JX, Zhang WX, Zheng GH, Zheng LW, Li ZM, Ge L. Application of "Liuzijue" breathing exercise in pulmonary rehabilitation of patients with stable COPD. J Fujian Univ Tradit Chin Med. (2008) 18:3-4.
17. Chen JX, Deng LJ. Rehabilitation effect of traditional "Liuzijue breathing exercise" on patients with stable COPD. Chin J Rehabil Med. (2009) 24:944-5.
18. Chen F. Effect of enhanced Liuzijue exercise on quality of life in patients with stable chronic obstructive pulmonary disease in plateau regions. J Plateau Med. (2024) 34:40-3.
19. Deng LJ, Zhang WX, Chen JX. Comparative study on the effects of Liuzijue and whole-body breathing exercise on respiratory function in elderly patients with chronic obstructive pulmonary disease. J Rehabil. (2018) 28:57-61.
20. Deng LJ, Chen JX, Chen YN, Fan LM, Zhou HJ, Chen FX. Study on the effect of Liuzijue exercise in elderly patients with chronic obstructive pulmonary disease with lung qi deficiency syndrome. China Med Innov. (2020) 17:112-7.
21. Fang DP, Liu Y, Zhu XL, Chen JX, Deng LJ, Tang AZ, et al. Application and effects of six-character formula respiratory gymnastics in continuous nursing for stable patients with COPD. J Nurs Manag. (2012) 12:813-5.
22. Fang Y, Fang Q, Wang SQ, Xu YK, Li Q, Chen M. The effects of simplified six-word formula combined with extracorporeal phrenic pace-making on SGRQ score, 6MWD and quality of life of patients with COPD stable period. Guangdong Med J. (2022) 43:1551-6.
23. Hu JB, Wei L, Tu HB, Yang PL, Wang ZW, Ji SQ. Rehabilitation effect of water-based Liuzijue for patients with stable chronic obstructive pulmonary disease. J Pract Clin Med. (2021) 25:45-7.
24. Ji SQ, Luo GW, Shen M, Liu XD, Yang PL, Wang ZW. Clinical study on Six-character formula in the intervention for COPD patients at stable phase. Liaoning J Tradit Chin Med. (2019) 46:1428-31.
25. Lan Y, Han X, Wang YY, Deng W, Liu SB, Feng YC. Impacts of the combined therapy of tiotropium bromide and medical exercise of the six-character formula on the living quality and pulmonary function in the patients of chronic obstructive pulmonary disease at the stable stage. World J Integr Tradit Chin West Med. (2016) 11:1369-71.
26. Li P, Liu J, Lu Y, Liu X, Wang Z, Wu W. Effects of long-term home-based Liuzijue exercise combined with clinical guidance in elderly patients with chronic obstructive pulmonary disease. Clin Interv Aging. (2018) 13:1391-9. doi: 10.2147/CIA.S169671.
27. Liu X, Wu W, Li N, Li P, Wang Z, Shan C. Effects of water‐based Liuzijue exercise on peak exercise capacity, functional exercise capacity, and quality of life in people with COPD. Clinical Respiratory J. (2021) 15:956–66. doi: 10.1111/crj.13399.
28. Lu F, Wang SC. Multicenter randomized controlled clinical study of "Liuzijue" breathing exercise for the treatment of stable COPD. Fujian J Tradit Chin Med. (2021) 52:1-3.
29. Shen Q. A community intervention study on treating COPD stabilization (level I) in elderly patients by Six strategics health exercises. J External Ther Tradit Chin Med. (2017) 26:3-6.
30. Shi XL, Ji SQ, Jiang FY, Yang PL, Wang ZW. Study on clinical effect of Six-character formula on patients with COPD in stable phase. Liaoning J Tradit Chin Med. (2020) 47:103-6.
31. Sun N, Li DP, Sun Y. Effect of Liuzijue combined with pursed-lip and abdominal breathing training on pulmonary function and quality of life in patients with stable COPD. Med Clin Res. (2019) 36:1003-4.
32. Wu W, Liu X, Li P, Li N, Wang Z. Effect of Liuzijue exercise combined with elastic band resistance exercise on patients with COPD: a randomized controlled trial. Evid Based Complement Alternat Med. (2018) 2018:2361962.
33. Wu W, Liu X, Liu J, Li P, Wang Z. Effectiveness of water-based Liuzijue exercise on respiratory muscle strength and peripheral skeletal muscle function in patients with COPD. Int J Chron Obstruct Pulmon Dis. (2018) 13:1713–26. doi: 10.2147/COPD.S165593.
34. Xiao C, Zhuang Y. Efficacy of Liuzijue Qigong in Individuals with Chronic Obstructive Pulmonary Disease in Remission. J Am Geriatr Soc. (2015) 63:1420–5. doi: 10.1111/jgs.13478.
35. Zhang FR. Rehabilitation nursing effect of traditional "Liuzijue breathing exercise" in patients with stable COPD. China Med Guide. (2019) (1):248.
36. Zhu Z. Health Qigong’s effect on the 61 stabilized patients with chronic obstructive pulmonary disease. J Nanjing Univ Tradit Chin Med. (2011) 27:235-7.
37. Cheng YF, Wei SS, He R. To observe the clinical curative effect of the traditional hua tuo style stable patients with chronic obstructive pulmonary disease. J Clin Chin Med. (2015) 27:683-5.
38. Gao YF, Qu YY, Chen MY. Effect of five-animal exercises on the lung function and exercise tolerance of chronic obstructive pulmonary disease patients during discharge transition period. J Clin Pathol. (2017) 37:975-80.
39. Liu FY, Tan PH. Application of community-based Wuqinxi exercise in patients with stable chronic obstructive pulmonary disease. J Henan Med Coll. (2020) 32:522-4.
40. Wei SS, Cheng YF, He R. The traditional Hua Tuo Wuqinxi in stable patients with chronic obstructive pulmonary disease effect of pulmonary function. J Clin Chin Med. (2015) 27:793-5.
41. Zhao QL. Clinical research on the prevention and cure stable phase chronic obstructive pulmonary disease in community by Five-animal exercises. Acta Chin Med. (2015) 30:801-3.
42. Zhu Y, Li N, Jin HZ. Effect on chronic obstructive pulmonary disease patient in stable condition by health qigong Wuqinxi exercise early treatment. J Liaoning Univ Tradit Chin Med. (2010) 12:107-10.
43. Chen JX, Deng YF, Chen Q, Zhang WX, Deng LJ. Effect of the third form of Baduanjin on quality of life and exercise endurance in patients with chronic obstructive pulmonary disease with lung-spleen qi deficiency syndrome. J Rehabil. 2015;25(3):13-7.
44. Chen X, Fu C, Wang X, Sun M, Shi J, Zhang W, et al. Combined Effect of 12 Weeks Baduanjin and Tri-Ball Respiratory Training as a Home-Based Pulmonary Rehabilitation in Subjects With Moderate Chronic Obstructive Pulmonary Disease: A Multi-Center Randomized Controlled Trial. Rehabil Nurs. (2025) 50:78–87. doi: 10.1097/RNJ.0000000000000493.
45. Chen Y, Zhang P, Dong Z, Zhu Y, Liu Y, Qiao C, et al. Effect of Baduanjin exercise on health and functional status in patients with chronic obstructive pulmonary disease: a community-based, cluster-randomized controlled trial. npj Prim Care Respir Med. (2024) 34:43. doi: 10.1038/s41533-024-00400-y.
46. Deng YF, Chen JX. Effect of the single-arm-lift form of Baduanjin on the rehabilitation outcomes of patients with chronic obstructive pulmonary disease. Chin J Nurs. (2015) 50:1458-63.
47. Deng W, Yang LL, Dong HZ, Zhang W. Evaluation of the efficacy of Deng’s Health-Preserving Baduanjin in pulmonary rehabilitation of patients with chronic obstructive pulmonary disease. Massage Rehabil Med. (2020) 11:41-4.
48. Dong LJ, Liu B. Effect of Shaolin Baduanjin on BODE index and quality of life in patients with stable chronic obstructive pulmonary disease. J Clin Chin Med. (2018) 30:1465-7.
49. Feng YC, Pan HS, Wen X, Chen CR, Jiao RY. Observation on the therapeutic effect of Baduanjin exercise in elderly patients with stable chronic obstructive pulmonary disease. New Chin Med. (2009) 41:36-7.
50. Feng LY, Zhang WJ, Huang JJ, Li DZ, Wei DQ, Lin DY, et al. Effect of Baduanjin training on lower limb muscle strength and quality of life in patients with chronic obstructive pulmonary disease. Shenzhen J Integr Tradit Chin West Med. (2024) 34:129-31.
51. Guo JC, Gao YF, Xie HX, Fang SR, Chen GY. Effect of Baduanjin exercise on rehabilitation outcomes in patients with stable chronic obstructive pulmonary disease. Qilu J Nurs. (2016) 22:97-8.
52. Guo XJ, Cao C, Chen N, Yan X, Zhang HL. Effect of Baduanjin exercise on extrapulmonary manifestations in community-dwelling elderly patients with stable chronic obstructive pulmonary disease. Chin J Pract Nurs. (2016) 32:2291-5.
53. Guo JC. Efficacy analysis of traditional Baduanjin exercise in promoting pulmonary rehabilitation in patients with stable COPD. J Shandong Med Coll. (2016) 38:171-4.
54. Huang BJ, Yao QP, Zhu YM. Observation on the efficacy of health Qigong Baduanjin as an adjunctive treatment for stable chronic obstructive pulmonary disease with lung-spleen deficiency syndrome. Hubei J Tradit Chin Med. (2017) 39:4-6.
55. Jiang X, Yang YR, Liu T, Wang BQ, Hua WS. Effect of traditional Baduanjin exercise on pulmonary rehabilitation in patients with stable chronic obstructive pulmonary disease. Chin J Health Care. (2023) 41:62-4.
56. Lei CY, Ye XC, Ji WJ, Wang JZ. Effect of Baduanjin exercise on exercise endurance and quality of life in patients with stable chronic obstructive pulmonary disease with lung-kidney qi deficiency syndrome. Chin J Rehabil Med. (2020) 35:992-4.
57. Liang XL. Effect of Baduanjin single-arm-lift rehabilitation exercise on the recovery of patients with chronic obstructive pulmonary disease. Nurs Pract Res. (2016) 13:156-7.
58. Liu SR, Chen YF. Clinical study on the effect of health Qigong Baduanjin on improving 6-minute walking distance in patients with stable chronic obstructive pulmonary disease. Sichuan Med J. (2013) 34:1090-2.
59. Liu GL. Effect of Baduanjin exercise on patients with stable chronic obstructive pulmonary disease. J Shandong Med Coll. (2021) 43:389-91.
60. Pan MY, Luo JH, Yang SC. Observation on the therapeutic effect of standing Baduanjin rehabilitation exercise in patients with stable chronic obstructive pulmonary disease. J Chengdu Univ Tradit Chin Med. (2016) 39:49-52.
61. Wang L, Fang L. Effect of practicing Baduanjin on pulmonary function in patients with stable chronic obstructive pulmonary disease. J Tradit Chin Med Guide. (2018) 24:86-7.
62. Wu Y, Yang JQ. Effect of Baduanjin combined with traditional Chinese medicine on respiratory function in patients with chronic obstructive pulmonary disease. Chin J Geriatr. (2019) 39:326-9.
63. Wu CY, Zhang HY. Observation on the effect of Baduanjin exercise in the rehabilitation treatment of patients with stable chronic obstructive pulmonary disease. Chin Prim Health Care Pharm. (2020) 27:2577-81.
64. Wu D, Yin ZF, Chen XB, Xu GX. Effect of remote home-monitored Baduanjin exercise on patients with chronic obstructive pulmonary disease. Chin J Rehabil Med. (2024) 39:628-33.
65. Yang BY. Application of Baduanjin-based lung, spleen, and kidney tonifying method as adjunctive therapy in patients with COPD. Int J Nurs. (2016) 35:2357-9.
66. Yang DL, Wu JH, Han ZJ. Evaluation of the efficacy of health Qigong Baduanjin in pulmonary rehabilitation of patients with stable chronic obstructive pulmonary disease. Chin Gen Pract. (2017) 20:356-8.
67. Ye XP. Effect of Baduanjin exercise on quality of life in elderly patients with stable chronic obstructive pulmonary disease. Contemp Nurse (Mid-month Ed). (2016) 4:80-2.
68. Yin XF. Study on the role of Baduanjin in the intervention and treatment of community-based patients with stable chronic obstructive pulmonary disease. Contemp Med Forum. (2024) 22:162-5.
69. Yu YY. Evaluation of the efficacy of health Qigong Baduanjin in pulmonary rehabilitation of patients with stable chronic obstructive pulmonary disease. World Latest Med Inf. (2019) 19:210-1.
70. Yu P, Jiang XF. Effect analysis of standing Baduanjin exercise in patients with stable COPD. Int J Nurs. (2021) 40:493-6.
71. Zhang LX, Wang T, Shi L, Zou Q, Zhu TG, Sun YL, et al. Effect of Baduanjin "regulating ancestral qi" on nutritional status and cardiopulmonary rehabilitation assessment in patients with chronic obstructive pulmonary disease. J Changchun Univ Tradit Chin Med. (2017) 33:954-6.
72. Zhang TT, Ma XQ, Chen RH, Yang Y. Evaluation of the effect of regular Baduanjin exercise on pulmonary function, fatigue status, and activity tolerance in patients with stable COPD. World J Integr Tradit Chin West Med. (2019) 14:415-8.
73. Zhu ZG, Chen Y. Effect of seated Baduanjin exercise on pulmonary function in patients with COPD. World J Integr Tradit Chin West Med. (2014) 9:846-8.
74. Zhu ZG, Fang S, Liang BH, Lin J, Shi XX, Chen Y. Effect of seated Baduanjin exercise on airway inflammatory response in patients with severe stable COPD. J Nurs Manag. (2017) 17:55-7.
75. Gao Y. Study on the effect of health Qigong Yijinjing in promoting pulmonary rehabilitation in patients with stable COPD. J Chengde Med Coll. (2016) 33:307-9.
76. Zhang M, Xu GH, Li F, Luo CF, Meng DJ, Ji Y. Health Qigong Yijinjing promotes rehabilitation in patients with stable chronic obstructive pulmonary disease. Chin J Sports Med. (2016) 35:339-43.
77. Zhang M, Xv G, Luo C, Meng D, Ji Y. Qigong Yi Jinjing promotes pulmonary function, physical activity, quality of life and emotion regulation self-efficacy in patients with chronic obstructive pulmonary disease: a pilot study. J Altern Complement Med. (2016) 22:810–7. doi: 10.1089/acm.2015.0224.
78. Donesky-Cuenco D, Nguyen HQ, Paul S, Carrieri-Kohlman V. Yoga therapy decreases dyspnea-related distress and improves functional performance in people with chronic obstructive pulmonary disease: a pilot study. J Altern Complement Med. 2009;15(3):225-234. doi:10.1089/acm.2008.0389.
79. Gupta A, Gupta R, Sood S, Arkham M. Pranayam for Treatment of Chronic Obstructive Pulmonary Disease: Results From a Randomized, Controlled Trial. Integr Med (Encinitas). (2014) 13:26-31.
80. Ranjita R, Hankey A, Nagendra H R, Mohanty S. Yoga-based pulmonary rehabilitation for the management of dyspnea in coal miners with chronic obstructive pulmonary disease: A randomized controlled trial. J Ayurveda Integr Med. (2016) 7:158–66. doi: 10.1016/j.jaim.2015.12.001.
81. Kaminsky D A, Guntupalli K K, Lippmann J, Burns S M, Brock M A, Skelly J, et al. Effect of Yoga Breathing (Pranayama) on Exercise Tolerance in Patients with Chronic Obstructive Pulmonary Disease: A Randomized, Controlled Trial. J Altern Complement Med. (2017) 23:696–704. doi: 10.1089/acm.2017.0102.
82. Thokchom S K, Gulati K, Ray A, Menon B K, Rajkumar. Effects of yogic intervention on pulmonary functions and health status in patients of COPD and the possible mechanisms. Complement Ther Clin Pract. (2018) 33:20–6. doi: 10.1016/j.ctcp.2018.07.008.
83. Yudhawati R, Rasjid Hs M. Effect of Yoga on FEV1, 6-Minute Walk Distance (6-MWD) and Quality of Life in Patients with COPD Group B. Adv Respir Med. (2019) 87:261–8. doi: 10.5603/ARM.2019.0047.
